# Heterogeneities in utilization of antenatal care in Uttar Pradesh, India: the need to contextualize interventions to individual contexts

**DOI:** 10.1080/16549716.2018.1517929

**Published:** 2018-11-13

**Authors:** Sanjeev Sridharan, Amanda Pereira, Katherine Hay, Arnab Dey, Dharmendra Chandurkar, Scott Veldhuizen, April Nakaima

**Affiliations:** a The Evaluation Centre for Complex Health Interventions, St. Michael’s Hospital and University of Toronto, Toronto, Canada; b The Evaluation Centre for Complex Health Interventions, St. Michael’s Hospital and University of Waterloo, Waterloo, Canada; c Bill and Melinda Gates Foundation, Seattle, USA; d Sambodhi Research and Communication, Noida, India; e McMaster University, Hamilton, Canada;; f The Evaluation Centre for Complex Health Interventions, St. Michael’s Hospital, Toronto, Canada

**Keywords:** Inequities, maternal health, India, Uttar Pradesh, antenatal care, evaluation

## Abstract

**Background**: This paper explores the heterogeneities in antenatal care (ANC) utilization in India’s most populated state, Uttar Pradesh. Taking an intersectionality lens, multiple individual- and district-level factors are used to identify segments of any antenatal care usage in Uttar Pradesh

**Objective**: This paper seeks to understand the multilevel contexts of ANC utilization. The planning and programming challenge is that such knowledge of contextual specificity is rarely known upfront at the initial stages of planning or implementing an intervention. Exploratory data analysis might be needed to identify such contextual specificity.

**Methods**: Tree-structured regression methods are used to identify segments and interactions between factors. The results from the tree-structured regression were complemented with multilevel models that controlled for the clustering of individuals within districts.

**Results**: Heterogeneities in utilization of any ANC were observed. The multiple segments of ANC utilization that were developed went from a low utilization of 23.7% for respondents who were not literate and did not have home ownership to a high of 82.4% for respondents who were literate and at the highest level of wealth. Key variables that helped define the segments of ANC utilization include: woman’s literacy, ownership of home, wealth index, and district-level sex ratio. Based on the multilevel model of any ANC utilization, cross-level interactions also were obtained between sex ratio and ownership of home as well as between sex ratio and literacy. Increases in sex ratio increased the influence of ownership of home on any ANC, while increases in sex ratio reduced the impact of woman’s literacy on receiving any ANC.

**Conclusion**: We argue that a focus on heterogeneous segments of utilization can help build knowledge of the mechanisms that underlie inequities in maternal health utilization. Such knowledge of heterogeneity needs to be incorporated in contextualizing interventions to meet a variety of recipients’ needs.

## Background

The social determinants of health provide a useful framework to understand how a range of factors can influence health and health equity outcomes [1,2]. The Commission for the Social Determinants of Health (CSDH) [1] recommends a focus both on structural determinants and on contextual specificities [1]. One important implication of taking a social determinants of health perspective is a movement away from a ‘one-size-fits-all’ policy approach to policies that are attentive to individuals’ differing contexts [1, p. 54].

While the focus of CSDH is at the policy level, the concerns about contextual specificities can also extend to the individual level [3,4]: How can health equity interventions respond to the contextual specificities in addressing the needs of individuals? For example, how can programmes respond to the empirical observation that different socio-economic groups utilize health systems at very different rates? In this paper we explore the heterogeneities of antenatal care (ANC) utilization in India’s most populated state, Uttar Pradesh. We focus on developing segments [5,6] of *any* antenatal care usage in Uttar Pradesh, with the objective that policy makers and programme planners use this information to serve women with low rates of ANC utilization. Any antenatal care usage [7] provides a measure of even a minimum level of engagement with the health system. Receipt of antenatal care marks the initiation of receipt of health services across the continuum of care and is an important outcome because it can drive other maternal health outcomes [7].

Our interest in this paper is to explore the interactions between different factors that are predictive of any antenatal care utilization in Uttar Pradesh. Most of the social determinants literature has not discussed the interactions between the multiple determinants and how these interactions might affect health care utilization. The findings in this study indicate that these interactions matter because the interplay between different factors helps explain why some individuals do not utilize the health system [8].

This paper utilizes data that were collected as part of an evaluation of an intervention called the Uttar Pradesh Technical Support Unit (UP-TSU) that seeks to build technical capacities of the state government of Uttar Pradesh in India to address maternal health problems. In 2012, the Bill and Melinda Gates Foundation and the government of Uttar Pradesh came together to form a Technical Support Unit that would provide techno-managerial support to the government in order to enhance its capacity to improve the coverage of reproductive, maternal, newborn, child health and adolescent health (RMNCH+A) interventions. to decrease mortality and enhance health.

The evaluation is informed by a realist evaluation perspective [3,9–11]. Realist evaluators argue that the programme impacts are a result of the programme context and mechanisms. This paper seeks to understand the context that this intervention aims to disrupt by developing a better understanding of the intersections of factors involved in any antenatal care utilization. Informed by a realist lens, we use a multilevel [12] framework to understand antenatal care utilization. Both district- and individual-level factors are considered in developing the segments of utilization. Our interest in such interactions is related to the growing discourse on intersectionality. Intersectionality is defined by Bowleg [13] as ‘a theoretical framework for understanding how multiple social identities such as race, gender, sexual orientation, socio-economic status, and disability intersect at the micro level of individual experiences to reflect interlocking systems of privilege and oppression.’ From an intersectionality perspective, critical guiding questions include: By what mechanisms does the programme intend to disrupt existing patterns of ‘synergies of oppression’? What specific resources does the programme bring to change the behaviours of individuals experiencing ‘intersections’ of disadvantage? Examining the segments help with developing an understanding of the multilevel contexts in which inequities need to be addressed.

**Figure 1.**
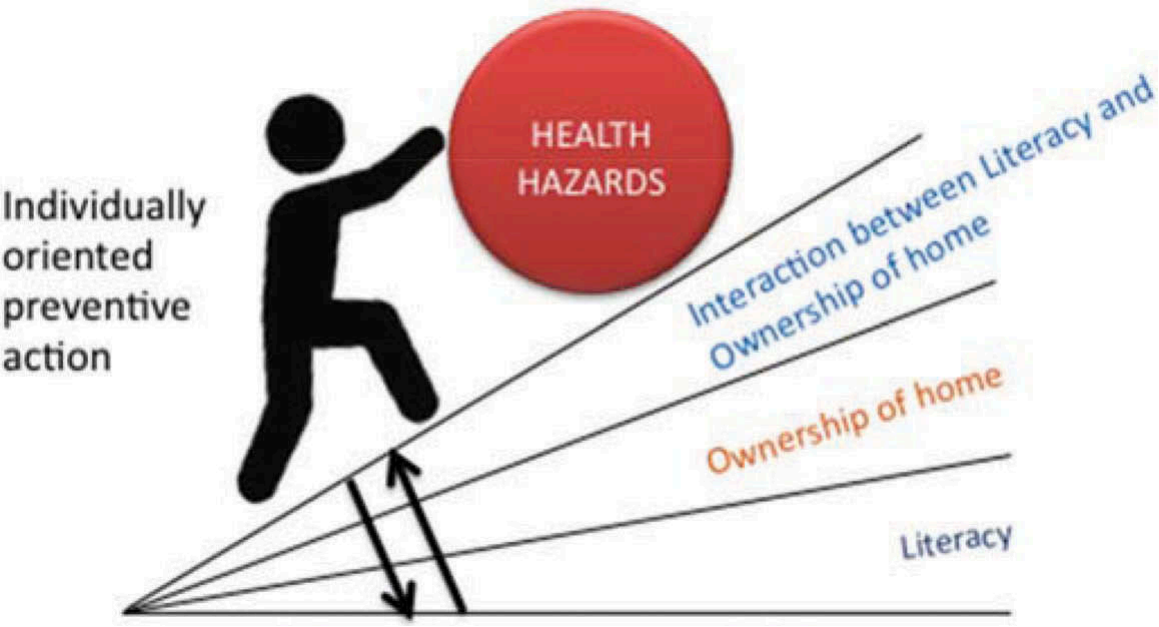
Example of direct and interaction effects of social determinants on health gradient.

**Figure 2.**
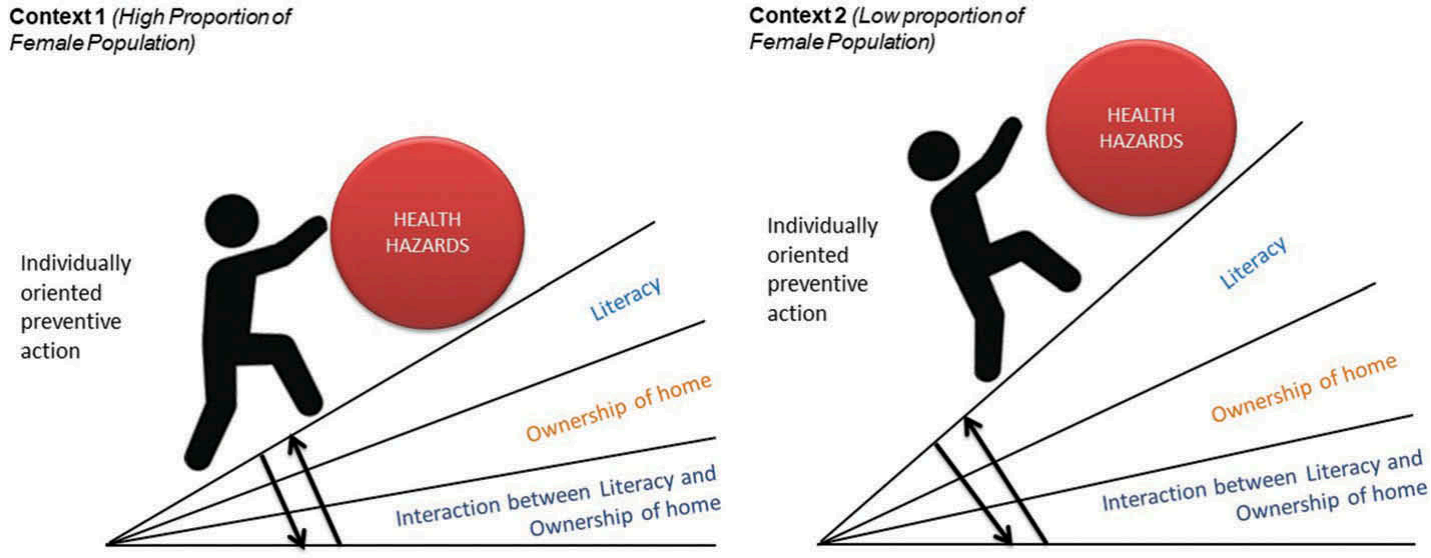
How context can potentially moderate the influence of the social determinants of health on the health gradient: a hypothetical illustrative example.

The theoretical ideas that provide justification for our focus on exploring intersections involved in utilization are discussed in Figures 1 and 2. Figure 1 describes the idea of a health gradient [14] that might result owing to the additive effects of multiple social determinants as well as the interaction between the social determinants. Note that the health gradient increases as a cumulative effect of different determinants as well as the interactions between the multiple social determinants of health. As a concrete example, in the context of maternal health utilization in India, consider the following: while being from a lower caste or being extremely poor can create disadvantages in terms of health outcomes, being simultaneously poor and from a lower caste can result in interaction effects that disproportionately reduce the likelihood of accessing maternal health services. This interaction additionally may be moderated by the community context – hence it is important to explore if the interaction differs across multiple contexts.

Figure 2 represents the idea that the combined cumulative impact of variables on health inequities might differ in different contexts. Our interest in incorporating factors at both individual- and district-levels is to better understand the contextual specificities in which a woman seeking antenatal care is located. Context is more than just a unitary dimension and can often encompass a conjunction of multiple factors across multiple levels [15]. The programming challenge is that such knowledge of contextual specificity is rarely known upfront at the planning of the intervention. Exploratory data analysis might be needed to identify such contextual specificities. For example, consider the complex relationship that might exist between education and district sex ratios. The relationship between a district sex ratio and the literacy rate in India follows a nonlinear, inverted U-shaped pattern, as a result of the double role played by education in the Indian context [16]. Education plays a positive role in reducing gender inequality by instilling in people values that do not give preference to either sex; conversely education can further the disparity in sex ratios by increasing an individual’s autonomy to use prenatal sex-detection technologies [17]. The contextual complexities that are present in a given individual’s circumstance highlight the importance of examining a problem from an intersectional lens as multiple individual and community-level factors can converge and influence various choices and decisions.

The state of Uttar Pradesh (UP) is an especially relevant setting to raise questions around antenatal care utilization. UP, India’s most populated state, has low rates of antenatal care utilization [18]. Only 7% of women in UP receive the four recommended antenatal care visits, and 85% of pregnant women give birth at home [18]. UP has a maternal mortality rate of 285 deaths for every 100,000 live births, which is much higher than the all India mortality rate of 167 deaths per 100,000 births [19]. The low rates of ANC utilization in this state as compared with some other parts of India stress the importance of examining the context and the need for identifying heterogeneous segments of utilization.

A number of individual-level contextual factors were considered in developing the segments including religion [20], caste [21,22], economic status [21–24], woman’s autonomy [21,25], literacy [21,22,24], husband’s education [21,22,24], age of marriage [21,26], and woman’s occupational status [21].

## Measures and analysis

### Sampling

The data used for the analysis are from a broader community-based evaluation study conducted in 49 districts in UP, India. The design of the study is described elsewhere in Sridharan et al. [12]. Participants sampled for the study included women with children 1 year of age at the time of the interview [*N* = 5,666] and were interviewed at their households.

### Measures

Informed by the social determinants of health, both district- and individual-level measures were included in developing the segments (see Table 1).

**Table 1.** Descriptive statistics for dependent and independent variables.

Variables	*N*	Percentage or mean	SD
**Dependent variables**			
Receipt of any antenatal care checkup (%, SD)	5,666	50.76%	0.50
**District-level measures**			
Sex ratio in district (mean, SD)	49	910.86	34.29
Proportion of marginalized workers in the district (%, SD)	49	33.06%	10.54
Proportion of illiterate population in the district (cannot read and write) (%, SD)	49	45.10%	6.72
**Individual-level measures**			
Respondent’s religion in Hindu (%, SD)	5,666	82.07%	0.38
Respondent belongs to Schedule Caste/Scheduled Tribe (SC/ST) (%, SD)	5,666	29.63%	0.46
Respondent belongs to Other Backward Classes (OBC) (%, SD)	5,666	57.17%	0.49
Respondent owns a house (%, SD)	5,666	84.75%	0.36
Respondent or any member of the her household have a bank account (%, SD)	5,666	88.63%	0.32
Respondent makes her own decision about her health care (%, SD)	5,666	60.36%	0.49
Respondent is allowed to go to the Health Centre on her own (%, SD)	5,666	18.81%	0.39
Respondent is literate (can read and write) (%, SD)	5,666	43.17%	0.50
Respondent had a job in the last 1 year (%, SD)	5,666	7.25%	0.26
Respondent was married at an age of 15 years or lower (%, SD)	5,666	61.33%	0.49
Respondent ever attended school (%, SD)	5,666	46.59%	0.50
Husband of the responded ever attended any school (%, SD)	5,666	75.40%	0.43
Number of members per sleeping room in the household (mean, SD)	5,666	0.39	0.30
Respondents age at the time of interview (mean, SD)	5,666	26.64	4.65
Wealth Index of the household^a^ (mean, SD)	5,666	0.01	3.02

^a^The wealth index is calculated for each household by combining a number of crucial household level assets and household characteristics. Principal component analysis was used to develop a continuous wealth index score.

District-level measures include: district sex ratio; proportion of the district farmers who are marginalized; and proportion of the district population who are not literate. Individual-level variables include: information on religion and caste; socio-economic status; measures of woman’s autonomy to make health care decisions; educational measures related to both the woman’s literacy and the husband’s schooling; other demographic variables including the age of the woman at the time of the interview as well as her age at first marriage – a binary variable measured if age at first marriage involved a child marriage (age equal to or less than 15 years). Sex ratio was calculated for the entire population in the 49 sampled districts in the state. It represents the total number of females per 1,000 men a particular district. The variable was computed from district-level data available from Census of India 2011.

### Analysis

#### Classification and regression trees

As a first step, segments of utilization are identified using classification and regression trees (CART) [27, 28]. These trees help suggest interactions between some of the social determinants. These approaches use recursive partitioning [28] techniques to cluster the data into homogeneous sub-groups that are distinct from each other based on a targeted response variable – in this application, receipt of *any* antenatal care.

CART analysis is a data-mining method used in classification problems. Typically, there is an outcome with two possible values, and the tree attempts to identify rules that sort individual cases into different segments. CART has been used to gain insight into risk factors and predictors for numerous outcomes, including outcomes related to colon cancer [29], nutrition [30], and cardiac mortality [31]. The goal within each of the segments is statistical homogeneity – the CART approach seeks to maximize within-node homogeneity [32–34]. The Gini impurity measure [33] is applied in our application.

Compared with methods more commonly used in epidemiological and medical research, trees have important advantages and disadvantages. Trees are non-parametric, with no assumptions about individual or joint variable distributions. They provide clearer results for developing segments than other kinds of models; a tree consists of an easy-to-understand set of decision rules that can produce a yes-or-no answer, making them particularly well suited to issues of clinical decision-making. They are also very good at identifying unsuspected interactions in the data: if certain combinations of factors strongly affect the probability of the outcome, this will be quickly apparent in the results. Importantly, CART can identify very complex interactions which are very unlikely to be noted in linear models, for example. There are, however, several important drawbacks to CART. The real challenge is being able to use it for causal analysis. We reiterate that CART is especially suited for exploratory analysis: our goal is not to use CART for inference but to develop ‘initial’ segments based on both the individual- and district-level factors.

#### Multilevel models

The descriptive classification and regression tree results have been complemented with the running of multilevel regression models [35] with interaction terms included. These models help confirm whether the interactions observed in the CART can be validated using alternative methodological approaches. These models are versatile in handling clustering of data [35]. Both district-level and individual-level information are included as predictors in the multilevel model. As the variable antenatal care usage was dichotomous, multilevel logistic regression models [35] are used in developing the models. The multilevel models are developed sequentially in two steps. The first set of models only explores the direct district- and individual-level factors associated with any antenatal care usage. Based on the results of the tree-structured regression, we include the cross-level interaction terms [35] for the second step of models between district and individual factors. Given space constraints, only the results of the second step are presented in this paper. Please note that the test statistic is the *t*-ratio formed by dividing the estimated regression coefficient by its standard error

## Results

### Identifying segments of utilization

The average antenatal care for our sample was 50.8%. The total sample size was 5,666 women. Of the 5,666 women surveyed, 50.8% (2,876 women) reported receiving any antenatal care. Key variables that were found to be important in the CART analysis included: literacy (whether the woman was able to read or write), wealth index, home ownership, age at time of interview, and district-level sex ratio (number of women to number of men residing in that district).

Literacy was the single variable that best discriminated between users and non-users of antenatal care: among women who could read or write, 62% sought antenatal care, compared with 42% among those who could not read or write.

**Figure 3.**
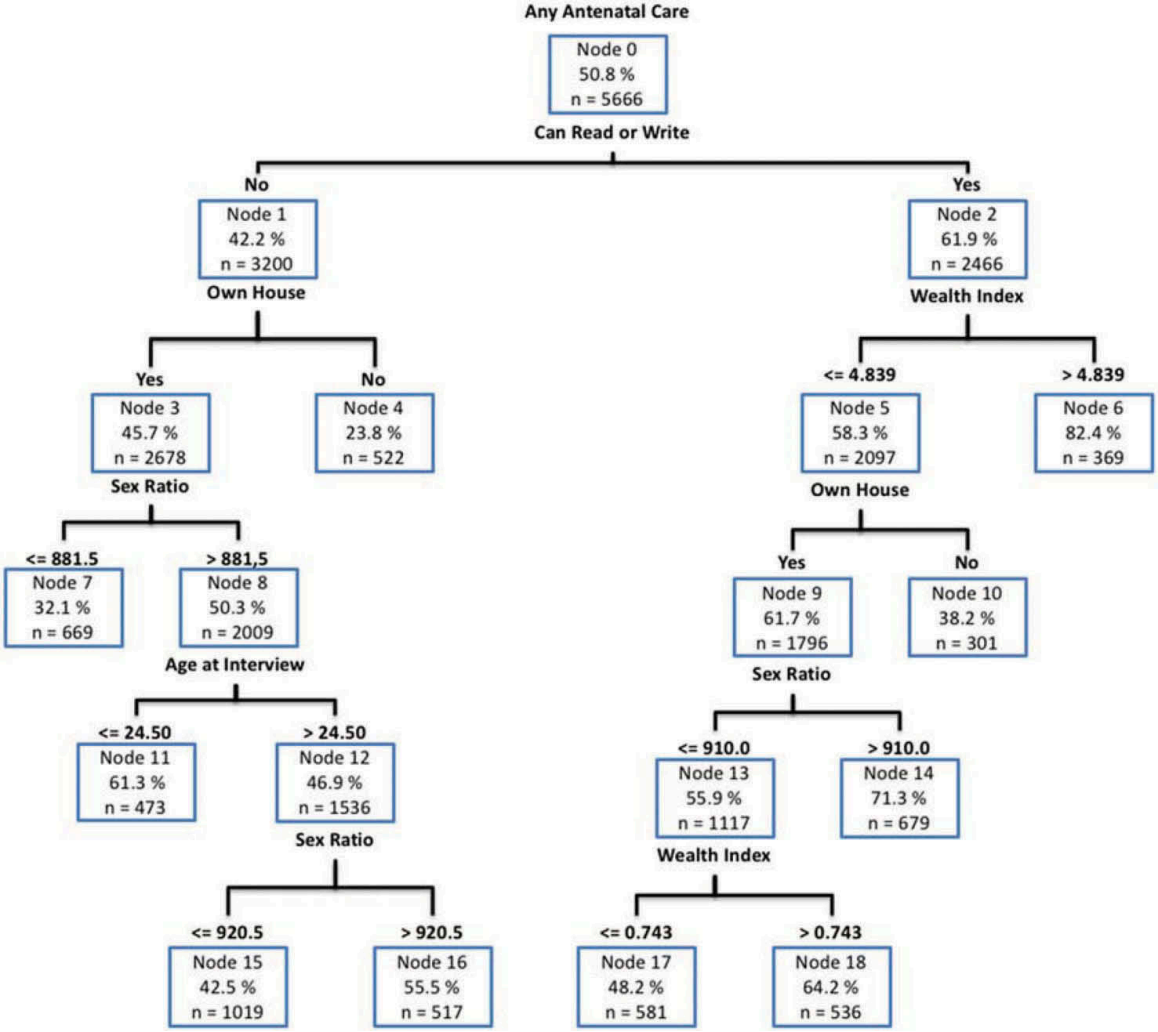
Tree diagram of any antenatal care usage in Uttar Pradesh.

We present the results both in this narrative and in the tree diagram below. Each branch of the tree breaks the results down further according to variables of literacy, wealth index, home ownership, etc. In the following narrative, we also describe the results for each of the tree branches. Figure 3 describes the obtained tree diagram.

As noted, for women who could read or write, the antenatal care usage rate increases to 61.9% (Node 2) from 50.8% for the total sample and 42% for those who could not read or write. This group is further split by the wealth index. For those in the highest values of the wealth index (wealth index greater than 4.84), the antenatal care rate increases to 82.4% (Node 6). For the wealth index below 4.84, any antenatal care usage rate is 58.3% (Node 5). Node 5 is further split by ownership of home. For those who do not own a home *and* whose wealth index is below 4.84 *and* who can read or write, the antenatal care rate drops to 38.2% (Node 10). However, for those who do own a home, antenatal usage rate rises to 61.7% (Node 9). Node 9 is further split by the variable ‘Sex Ratio.’ In districts where the sex ratio is greater than 910, the antenatal care usage rate increases to 71.3% (Node 14). However, in districts where the sex ratio is below 910, the antenatal care usage rate is 55.9% (Node 13). Node 13 is further split by the wealth index. For those whose wealth index is greater than 0.74, antenatal care usage is 64.2% (Node 18). However, for those whose index is below 0.74, antenatal care usage is 48.2% (Node 17).

Returning to the top of the tree, women who are unable to read or write have an antenatal care usage rate of 42.2% (Node 1). For those who do not read or write *and* also do not own a home, antenatal care utilization rate is 23.8% (Node 4). However, for those who do not read or write *and* do own a home, the antenatal care usage rate is 45.7% (Node 3). Node 3 is further split by sex ratio. In districts where the sex ratio is greater than 882, antenatal care usage rate increases to 50.3% (Node 8). For those in Node 3 who live in districts where the sex ratio is less than 882, antenatal care usage rate is 32.1% (Node 7). Node 8 is further split by the age at interview. For those above 24.5 years at the time of interview, antenatal care usage rate is 46.9% (Node 12); for those below or equal to 24.5 years, antenatal care usage rate is 61.3% (Node 11). This result indicates that in general older women are less likely to seek antenatal care than younger women. Node 12 is further split by sex ratio. In districts where the sex ratio is greater than 920.5, antenatal care usage rate is 55.5% (Node 16). In districts where the sex ratio is less than 920.5, antenatal care usage rate is 42.5% (Node 15).

**Figure 4.**
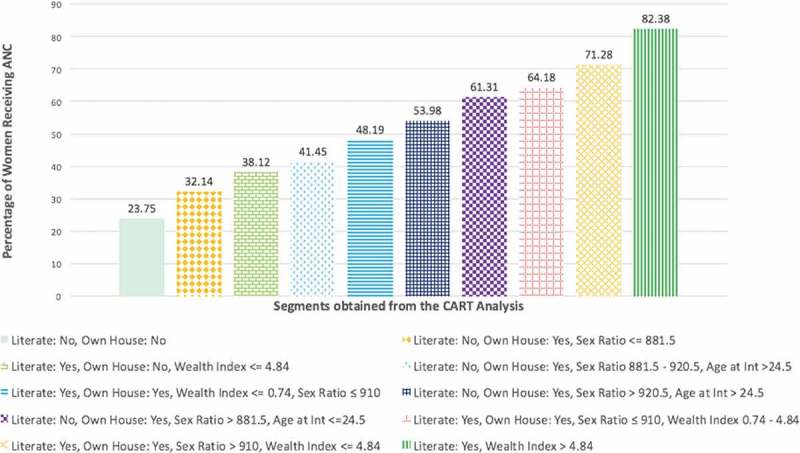
Heterogeneities in utilization of any antenatal care.

Figure 4 describes the heterogeneities in utilization of any ANC. It goes from a low of 23.7% for respondents who are not literate and do not have home ownership to a high of 82.4% for those who are literate and at the highest levels of wealth.

Much of the above results are descriptive and suggest patterns in the data without a clear theoretical specification. However, the above results also suggest interactions between sex ratio and the respondent’s literacy and ownership of a home. Based on the patterns observed in Figure 3 we also included a cross-level term between sex ratio and literacy and ownership of home. Table 2 describes the results of running the multilevel models. Key predictors include: caste (being in a scheduled caste or a scheduled tribe category reduces the odds of receiving any antenatal care by 21%); ownership of home (increases the odds of receiving antenatal care by 180%); literacy (increases the odds by 60%); any job in the last year (increases the odds of receiving any antenatal care by 35%); wedding below the age of 15 (reduces the odds of receiving any antenatal care by 29%); husband’s schooling (husband having schooling increased the odds of receiving any antenatal care by 29%); and wealth (increased wealth enhances the odds of receiving antenatal care). Cross-level interactions were also obtained between ownership of home and sex ratio as well as literacy and sex ratio. Increases in sex ratio increased the influence of ownership of home, while increases in sex ratio reduced the impact of women’s literacy on receiving any antenatal care.

**Table 2.** Results of the multilevel model with interaction terms included for receipt of any antenatal care checkup.

Variables	*B* (std. error)	Odds ratio	95% CI of odds ratio	*T*-ratio
**District-level measures**				
Sex ratio in district	−0.001 (0.004)	0.99	(0.99, 1.01)	−0.23
Proportion of marginalized workers in the district	0.012 (0.007)	1.00	(0.99,1.03)	1.66
Proportion of illiterate population in the district (cannot read and write)	0.004 (0.008)	1.01	(0.99, 1.02)	0.49
**Individual-level measures**				
Respondent’s religion in Hindu	−0.148 (0.098)	0.86	(0.71,1.05)	−1.51
Respondent belongs to Schedule Caste/Scheduled Tribe (SC/ST)	−0.230 (0.129)	0.79	(0.62,1.02)	−1.79
Respondent belongs to Other Backward Classes (OBC)	−0.312** (0.118)	0.73	(0.58, 0.92)	−2.63
Respondent owns a house	1.037*** (0.102)	2.80	(2.31, 3.45)	10.16
Respondent or any member of the her household have a bank account	0.117 (0.111)	1.12	(0.91,1.40)	1.06
Respondent makes her own decision about her health care	−0.091 (0.061)	0.91	(0.81,1.03)	−1.50
Respondent is allowed to go to the Health Centre on her own	−0.068 (0.075)	0.93	(0.81,1.08)	−0.91
Respondent is literate (can read and write)	0.470** (0.139)	1.60	(1.22,2.10)	3.39
Respondent ever attended any school	−0.099 (0.129)	0.91	(0.70, 1.17)	−0.77
Respondent had a job in the last 1 year	0.297* (0.119)	1.35	(1.07,1.70)	2.51
Respondent was married at an age of 15 years or lower	−0.337*** (0.065)	0.71	(0.63,0.81)	−5.18
Husband of the responded ever attended any school	0.256*** (0.063)	1.29	(1.14,1.46)	4.00
Number of members per sleeping room in the household	−0.189 (0.122)	0.83	(0.65,1.05)	−1.54
Wealth Index of the household	0.102*** (0.013)	1.11	(1.08,1.14)	8.22
Respondents age at the time of interview	−0.031*** (0.006)	0.97	(0.96,0.98)	−5.30
**Cross-level interactions**				
Ownership of home × sex ratio	0.009** (0.003)	1.01	(1.00, 1.01)	3.22
Literacy × sex ratio	−0.004* (0.002)	0.99	(0.99, 1.00)	−2.57

Level of significance: *p < 0.10, **p < 0.05, ***p < 0.001.

## Discussion

### Implications

This paper helps identify segments of any antenatal care utilization based on individual and district-level measures. As described in Figure 3 and Table 2, key variables that were found to be useful in defining segments included: literacy, ownership of home, wealth index, and district-level sex ratio. Additionally statistically significant interactions were observed between district-level sex ratio and literacy, and district-level sex ratio and ownership of home. The key result was the remarkable heterogeneity of utilization in the segments. Utilization varies between 23.7% and 82.4% across the segments. Further, the segments were defined by both individual- and contextual-level factors.

These results suggest that understanding the process by which disadvantage can accumulate needs to incorporate knowledge of ‘intersections’ between multiple levels. The important practical challenge this raises is that of the need for the intervention to adapt to the needs of the multiple segments. How can the intervention specifically respond to the differences in contexts, such as variations of sex ratios across different districts or approaches to reach women who do not read or write? Paying attention to the heterogeneity of segments is important because by not reaching individuals who are less likely to use the system, an intervention can exacerbate health inequities [4].

One important implication emerging from the results in this paper is the importance of considering multilevel interactions when thinking about intersections of oppression and privilege. Such a multilevel view is consistent with the eco-epidemiological approach [36]. This view is also consistent with a growing methodological focus on using multilevel models to include not only individual-level variables but also community level and other contextual levels to understand their cross-level interactions [36–40]. An unfavourable social context can challenge binary thinking as it brings light to the stark reality of people’s lives. One can experience both privilege and affliction concurrently [41]. For example, as depicted in Figure 2, the segment of women who have privileges such as owning a home and being literate but face disadvantages such as being poor and living in a district with an unbalanced sex ratio have an antenatal care usage rate of only 48.2%. This suggests that it is crucial to understand the interlocking systems of privilege and oppression that lead to any ANC utilization as opposed to examining this outcome solely from a binary lens (e.g. literacy vs. illiteracy).

Support for using a multilevel perspective on intersectionalities is obtained in the literature. A study on maternal health care utilization in nine high-focus states in India noted that for women who belonged to scheduled castes or scheduled tribes, it was the intersection of their low SES status, their segregated habitation, and their lack of knowledge about maternal care that led to under-utilization of maternal health care services [40]. Studies about maternal health care utilization in Nigeria and Mali [37–39] have stressed the importance of using a multilevel approach in understanding maternal health utilization [38]. These studies describe how higher levels of education interact with a host of pathways such as improved knowledge of health services and increasing autonomy at the individual level [39].

There are three implications of our results for further research. First, in UP, the sex ratio of a district was an important contextual factor that emerged in the analysis; districts with lower female-to-male sex ratios had lower utilization of ANC. It is important to probe the meaning of sex ratios in a rural Indian context. Low sex ratios are an indication of unfavourable life chances for females, especially in contexts where girls are missing owing to factors like sex selective abortion and neglect in childhood [42]. It is possible that the sex ratio might be a proxy for women’s empowerment and rights. Future qualitative research could play an important role in understanding how a society’s view of women’s rights and empowerment could intersect with her utilization of health care services. Qualitative work is needed to explore the relationship between sex ratio and community level measures of women’s empowerment to better explore this relationship. Second, the intersections between sex ratio and individual-level measures, such as literacy, need to be probed further through qualitative methods to better shed light on the mechanisms that may drive such intersections. Third, our choice of districts as one level of analysis was driven by availability of data. We think that going forward, future work needs to study the obtained relationships using finer levels of analysis, using the block and other finer units.

### The need for modifications in the programme over time

The segments described in this paper can help identify gaps in regard to ANC utilization. programmes aiming to target ANC uptake should focus their outreach at women who fall in these gaps. The dependence of intersectionalities on context [43,44] suggests that interventions have to adapt to suit the population they are attempting to serve. Thinking of interventions as dynamic systems [45,46] that evolve over time also is helpful in that it provides a direction in how the evaluation itself serves as a means of developing knowledge for further implementation and contextualization of the intervention. This example also highlights that the programme theory for a number of interventions that focus on inequities is often incomplete [46]. As example, knowledge of heterogeneities of utilization is often missing in the initial stages of implementing an intervention. Such knowledge of heterogeneities needs to be incorporated in contextualizing an intervention to meet a variety of needs [47]. Our view is that the data analysis has a role to play in identifying such segments, especially in the absence of a theory of change supported by strong and contextually relevant evidence.

### Limitations

Key limitations include that this paper is intended to be exploratory, to generate a series of questions that can help further the field around intersectionalities. The theoretical foundation of multilevel interactions in intersectionality research still needs to be developed further. Additionally, much research on intersectionality has been primarily theoretical. There still needs to be a broader dialogue around a range of measures and the different levels of measurement of various measures used to develop the segmentsthat can lead to understanding how interventions can address challenges of intersectionalities. The segments obtained in this paper are suggestive of underlying mechanisms that might be operating. The quantitative results in this paper need to be complemented with focused qualitative work that can highlight some mechanisms that generate the inequitable service utilization associated with these intersectionalities.

Additionally, we have already noted some of the challenges of recursive partitioning techniques, such as CART. A number of researchers insist on well-defined theoretical specifications in developing models. Our view is that theories can help identify the variables in a model specification, but the functional form of the variables (e.g. nonlinearities, interactions, thresholds, etc.) can be aided through data exploration.

## Conclusions

The analysis described in this paper raises a number of conceptual questions for future implementations and evaluations of maternal health initiatives. The Commission on Social Determinants of Health [1] contends that ‘Arguably the single most significant lesson of the CSDH conceptual framework is that interventions and policies to reduce health inequities must not limit themselves to intermediary determinants, but must include policies crafted to tackle structural determinants.’ As we have highlighted in this paper, there is a need to incorporate knowledge of structural determinants in planning responses to health inequities. Key questions that emerge from this paper include: How is the knowledge that can be gathered from the intersections of different categories of utilization rates used to design and improve programmes to better target the challenges of intersectionalities that are about tackling structural determinants? Given the important role of structural determinants such as education and wealth, how does one set realistic expectations of the types of impacts that interventions can have on impacting inequities? What kinds of leverage [48] do interventions such as the Technical Support Unit really have to address inequities? We think that a focus on heterogeneity of segments of maternal health utilization can help build knowledge of the mechanisms that underlie inequities in maternal health utilization.

## Paper context

Different groups of individuals use antenatal care services at different rates. This paper identifies a wide variation in antenatal care use and also the interaction between factors in such use. Understanding such diversity in antenatal care use is helpful in better understanding some of the barriers that individuals face in using the health system. It can also help with planning programmes and policies to address the wide gaps in utilization between different groups of individuals.
